# Quantitative hemodynamics of draining veins in brain arteriovenous malformation: a preliminary study based on computational fluid dynamics

**DOI:** 10.3389/fneur.2024.1474857

**Published:** 2024-12-11

**Authors:** Long Ma, Yu Chen, Pingting Chen, Li Ma, Debin Yan, Ruinan Li, Zhipeng Li, Haibin Zhang, Heze Han, Kexin Yuan, Runting Li, Fa Lin, Yuanli Zhao, Xiaolin Chen

**Affiliations:** ^1^Department of Neurosurgery, Beijing Tiantan Hospital, Capital Medical University, Beijing, China; ^2^College of Energy and Power Engineering, Nanjing University of Aeronautics and Astronautics, Nanjing, China; ^3^Department of Neurosurgery, Peking University International Hospital, Peking University, Beijing, China; ^4^Department of Neurosurgery, Peking Union Medical College Hospital, Chinese Academy of Medical Sciences and Peking Union Medical College, Beijing, China

**Keywords:** arteriovenous malformation, hemodynamic, angioarchitecture, computational fluid dynamics, wall shear stress, pressure

## Abstract

**Objective:**

This study initiated a preliminary computational fluid dynamics (CFD)-based study to investigate the relationship between quantitative hemodynamics of arteriovenous malformation (AVM) draining veins and rupture.

**Methods:**

The quantitative hemodynamics of AVM draining veins were generated from computed tomography angiography (CTA)-based steady-state CFD models. Morphological and hemodynamic parameters were compared between the ruptured and unruptured groups. The boundary conditions of the drainage vein were obtained from quantitative digital subtraction angiography (QDSA). The draining veins were divided into 15 consecutive segments to analyze the spatial distribution of the hemodynamic parameters by linear regression analysis.

**Results:**

From 11 AVMs, it was revealed that morphological parameters of drainage veins in ruptured and unruptured AVMs were similar. The intravascular pressure of the draining vein in the ruptured AVMs was significantly higher than those of the unruptured AVMs (pressure average: *p* = 0.006; pressure maximum: *p* = 0.045), and the WSS of the posterior segment was higher in ruptured AVMs (*p* = 0.045). WSS of draining veins in ruptured AVMs showed a linear increase trend with segmenting (*R* = 0.731, *p* < 0.001), and ruptured AVMs were more likely to be accompanied by high-velocity segments in the draining vein (40.0% vs. 14.7%, *p* = 0.037), especially in the posterior segment (*p* = 0.011).

**Conclusion:**

The draining veins of ruptured AVMs had significantly higher intravascular pressure and posterior segment WSS. WSS showed a linear increase with segmentation in ruptured AVMs, and they often had more high-velocity segments in the draining vein, especially in the posterior segment.

## Introduction

Brain arteriovenous malformations (AVMs) are tangles of abnormally dilated vessels without intervening capillaries, which represent high-flow and low-resistance hemodynamic features due to direct arteriovenous shunting (1). AVMs represent 38% of all intracerebral hemorrhage in patients between 15 and 45 years of age (2). The natural annualized rupture risk was estimated to range from 2–4% per year (3). Several studies have identified several angioarchitecture predictors of AVMs to cause intracranial hemorrhage (4–6). However, results were inconsistent due to the small sample size and wide-existing selective bias. Therefore, exploring the internal pathophysiology and biomechanical mechanism of AVM rupture is the objective solution to avoid the above bias.

In theory, specific angioarchitecture may result from long-term hemodynamic effects. Shakur et al. (7) and Chang et al. (8) suggested that variation in wall shear stress (WSS) between feeders and normal arteries relates to the clinical presentation in AVMs. However, a growing number of recent studies have highlighted the importance of draining veins in the mechanism of AVM rupture (9). The theory of occlusive hyperemia set forth by Al-Rodhan et al. (10) offers convincing evidence on the contribution of obstruction of venous drainage. Impairment of venous drainage has been shown to be significantly associated with hemorrhagic risk (11, 12), which indicates that the increased resistance in the draining veins may induce hemorrhage by pressurizing the AVM system. Computational fluid dynamics (CFD) has been employed to characterize the local hemodynamic features that contribute to the pathogenesis of cerebral vascular disease (13, 14). However, current research does not thoroughly explore the hemodynamic mechanisms of draining veins in AVM rupture by CFD analysis. Challenges include obtaining specific boundary conditions for these veins and their irregular shapes compared to arteries.

In this study, we initiated a preliminary CFD-based study to explore the hemodynamic characteristics of AVM-draining veins and their relationship with clinical manifestations.

## Materials and methods

### Study protocol approvals and patient consents

The study protocol was approved by the Institutional Review Board of Beijing Tiantan Hospital and was carried out according to the guideline of the Helsinki Declaration. Written informed consent was obtained from all participants and their guardians at admission.

### Study design and participants

**Figure 1:**
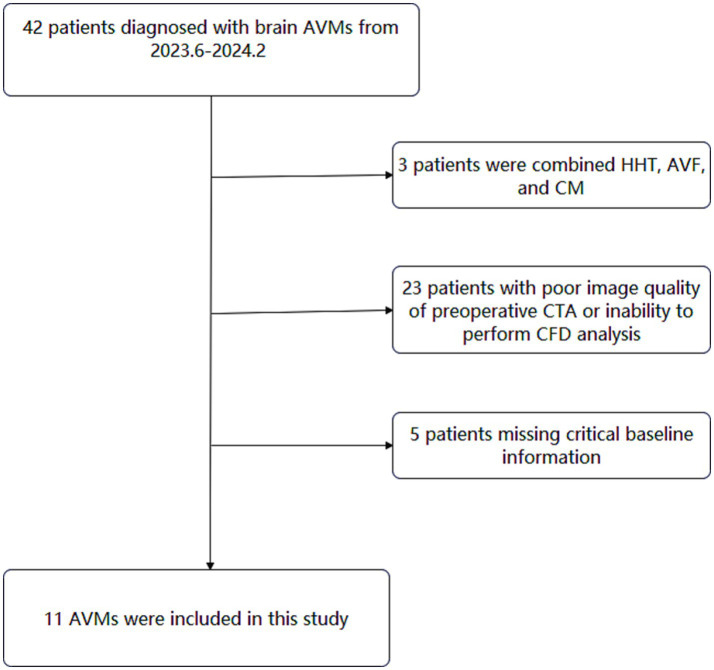
Flowchart of study participants.

This retrospective study reviewed 42 consecutive brain AVMs between June 2023 and February 2024 in a single tertiary referral institution (Beijing Tiantan Hospital, Figure 1). The inclusion criteria were as follows: (1) patients diagnosed with AVM by digital subtraction angiography (DSA), and the DICOM data of DSA and preoperative computed tomography angiography (CTA) were available; (2) patients with single draining vein (in cases involving multiple draining veins, the most dominant draining vein was identified and selected for analysis). (3) Patients with supratentorial nidus (avoid measurement errors caused by different circulation systems). Exclusion criteria were as follows: (1) patient’s concomitant diagnosis of hereditary hemorrhagic telangiectasia (HHT), arteriovenous fistula (AVF), and cavernous malformation (CM); (2) patients with poor image quality of preoperative CTA; (3) patients missing critical baseline information. After rigorous review, a total of 11 AVMs were included in this study.

### Baseline characteristics, vascular modeling and morphology parameters

Demographic, clinical, and imaging data were recorded. The hemorrhagic presentation was defined as hemorrhage that could be attributed to AVM rupture with symptomatic and CT evidence. The definition of morphological characteristics included AVM size, eloquent area, deep venous drainage, venous dilation, venous stenosis, feeding artery dilation, and flow-related aneurysm were based on the terminology associated with brain AVMs provided by a joint committee led by the American Society of Interventional and Therapeutic Neuroradiology (15).

CTA was performed following admission and the images were saved as the DICOM format and the generated models were exported in a stereolithography (STL) format. The data were then imported into Mimics 19.0 (Materialize, Belgium) to reconstruct 3-dimensional (3D) AVM structure for the following morphological-hemodynamic analysis. Morphological parameters were measured from the reconstructed 3D AVM model by two independent investigators (LoM and PC). The actual cross-sectional area of the inlet/exit was selected in the calculation of the outlet/inlet cross-sectional area ratio. To reflect the degree of tortuosity, we introduce the concept of tortuosity index, which is the ratio of the actual length of the 3D space (actual length) to the straight distance between the entrance and the exit (absolute length).


Tortuosity index=Actual length/Absolute length

### CFD simulation and hemodynamic parameters

Procedures of CFD modeling in 3D AVM models were as follows: the CTA images were exported in a stereolithography (STL) format. After vascular modeling by Mimics, the 3D models were segmented and smoothed by the software Geomagic Studio 2012 (North Carolina) and then imported into ICEM CFD 2019R3 (ANSYS, Lebanon, NH) to create volume grids for fluid dynamics simulation. The draining veins were divided equally into 15 segments according to the vascular centerline. In order to compare the distribution difference of hemodynamic parameters in drainage veins among different segments, the drainage vein was divided into 3 major segments based on the aforementioned 15-segment segmentation method, including segment A (segment 1–5), segment B (segment 6–10) and segment C (segment 11–15).

**Figure 2:**
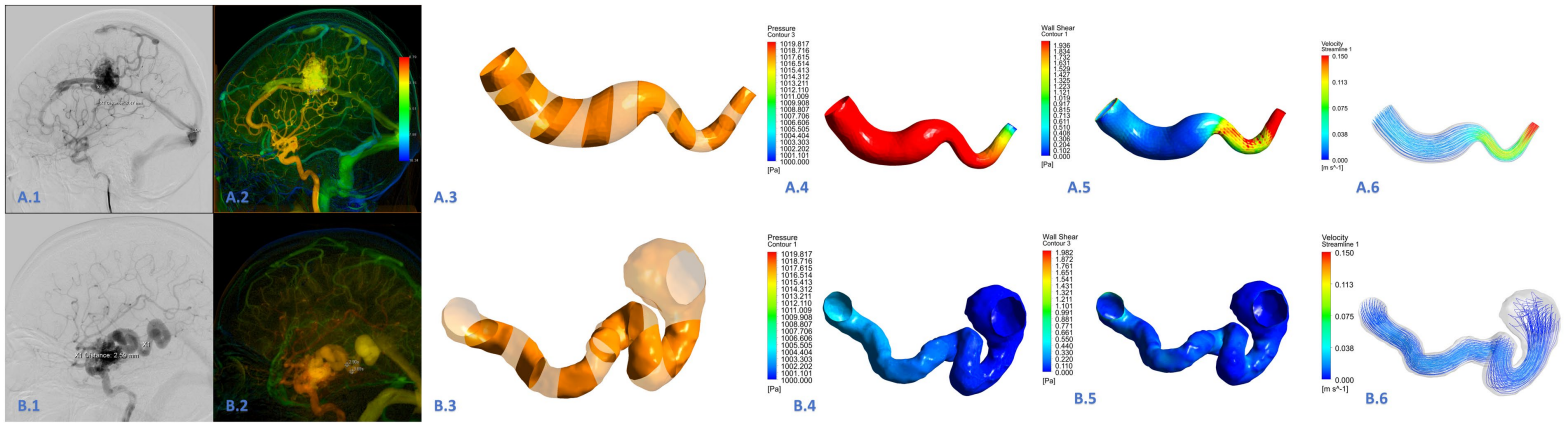
Hemodynamic analysis of the draining vein in the ruptured **(A.1–A.6)** and unruptured **(B.1–B.6)** AVMs. This was a draining vein in the ruptured AVM **(A.1,A.2)**. The draining vein was divided into 15 segments **(A.3,B.3)**. The wall pressure of the draining vein was high in all segments, the wall shear stress was high in the posterior position, and high blood flow velocity was common **(A.4–A.6)**. This was a draining vein in the unruptured AVM **(B.1–B.2)**. The wall pressure, wall shear stress, and blood flow velocity were generally lower than those in the draining vein of ruptured AVM **(B.4–B.6)**.

Previous studies did not provide information on the boundary conditions of the AVM draining veins. To bring the CFD simulations closer to reality, specific blood flow velocities at the proximal end of the draining vein were obtained by QDSA and DSA the ROI area was divided at the beginning of the draining vein, and the specific blood flow velocity was obtained by the distance through the ROI area/the time through the ROI area. All DSA procedures were performed using a single C-arm fluoroscopy system (Philips, Amsterdam, Netherlands). A standard angiographic technique and contrast medium injection method were applied to all patients. The schematic diagram is shown in Figure 2. The specific blood flow velocities were applied at the draining veins inlets. The pressure of 0 Pa applied at the draining vein’s outlet to mimic venous sinus pressure. CFD simulations were performed by ANSYS CFX 19.2 (ANSYS Inc.), The governing equations underlying the calculation were the Navier–Stokes formulations. The blood was assumed as an incompressible flow with a constant density of 1,066 kg/m^3^ and a dynamic viscosity of 0.0035 Pa s. For the arterial wall, it was assumed to be rigid, no-slip, and no-penetration conditions were imposed. Hemodynamic characteristics of the AVM draining veins in the CFD models were assessed in ANSYS CFD-post.

Hemodynamic parameters were measured simultaneously by two researchers (LoM and YC), including WSS and pressure. The draining veins were divided into 15 consecutive segments to analyze the spatial distribution of the hemodynamics. The pressure in each segment, pressure average, pressure maximum, WSS in each segment, WSS average, and WSS maximum were computed for each patient. Besides, the number of segments with high velocity was recorded to evaluate the degree of intravascular flow disorder. To intuitively evaluate the blood flow velocity and WSS level, the speed scale was set as 0–0.15 mm/s, the WSS scale was set as 0–2 Pa, and the pressure scale was set as 1,000–1,020 Pa, with blue representing low value and red representing high value. High velocity was defined as a flow rate exceeding 0.113 m/s, which corresponds to the highest speed range indicated by the color level.

### Statistical analysis

Statistical analyses were conducted with SPSS 25.0 (IBM Corporation, New York, United States) and GraphPad Prism 10 (GraphPad Software, CA, United States). The categorical data were expressed as numbers and percentages, and continuous data were shown as median and inter-quartile range. For the demographic and angioarchitecture data, ruptured and unruptured AVMs were compared using descriptive statistics. Categorical variables were expressed as frequencies, and continuous variables were described as mean with standard deviation (SD) or median with interquartile range (IQR). For the draining veins’ morphological and hemodynamic parameters, the Mann–Whitney *U* test (Wilcoxon rank-sum test) was applied. Besides, the correlation coefficients of quantitative hemodynamic parameters of draining veins in subgroups with different clinical phenotypes were calculated using the Spearman rank method. The strength of correlation was measured with the coefficient determination (*R*). The relationship with *R* > 0.50 or <−0.50. Statistical significance was set at *p* < 0.05.

## Results

### Baseline characteristics

The study cohort consisted of 11 patients with a median age of 26.0 (21.5, 31.5) years old. 5 (45.5%) were male, and 6 (54.5%) were ruptured. There were 1 (9.1%) Spetzler-Martin (SM) grade I, 3 (27.3%) SM grade II, 5 (45.5%) SM grade III, and 2 (18.2%) SM grade IV AVMs.

There were no significant differences in the angioarchitecture characteristics between the ruptured and unruptured AVMs. For the draining veins, both ruptured and unruptured group had 1 patient accompanied with stenosis (16.7, 20.0%, respectively), while both these two groups had 2 patients accompanied with venous aneurysm (33.3, 40.0%, respectively) (Table 1).

**Table 1:** Baseline characteristics.

Characteristics	All cases	Ruptured	Unruptured	*p*-value
No. of patients	11	6 (54.5)	5 (45.5)	
Sex, male (%)	5 (45.5)	2 (33.3)	3 (60.0)	0.376
Age [median (IQR)]	26.00 [21.50, 31.50]	26.50 [24.50, 31.50]	23.00 [20.00, 31.00]	0.410
Smoking (%)	4 (36.4)	2 (33.3)	2 (40.0)	0.819
Hypertension (%)	0 (0.0)	0 (0.0)	0 (0.0)	>0.999
Eloquent area (%)	5 (45.5)	2 (33.3)	3 (60.0)	0.376
AVM side, left (%)	5 (45.5)	2 (33.3)	3 (60.0)	0.376
Spetzler-Martin grade (%)				0.325
I	1 (9.1)	0 (0.0)	1 (20.0)	
II	3 (27.3)	1 (16.7)	2 (40.0)	
III	5 (45.5)	3 (50.0)	2 (40.0)	
IV	2 (18.2)	2 (33.3)	0 (0.0)	
V	0 (0.0)	0 (0.0)	0 (0.0)	
Lawton–Young grade (%)				0.152
4	3 (27.3)	1 (16.7)	2 (40.0)	
5	4 (36.4)	3 (50.0)	1 (20.0)	
6	2 (18.2)	0 (0.0)	2 (40.0)	
7	2 (18.2)	2 (33.3)	0 (0.0)	
Feeding artery dilation (%)	9 (81.8)	5 (83.3)	4 (80.0)	0.887
Single feeding artery (%)	2 (18.2)	1 (16.7)	1 (20.0)	0.887
Lesions diffuse (%)	4 (36.4)	3 (50.0)	1 (20.0)	0.303
Arteriovenous fistula (%)	1 (9.1)	0 (0.0)	1 (20.0)	0.251
Deep venous drainage (%)	5 (45.5)	3 (50.0)	2 (40.0)	0.740
Draining venous stenosis (%)	2 (18.2)	1 (16.7)	1 (20.0)	0.887
Venous aneurysm (%)	4 (36.4)	2 (33.3)	2 (40.0)	0.819

AVM, arteriovenous malformation. Values are expressed as number of cases (%) or median ± IQR, unless otherwise indicated. ^*^Statistical significance (*p* < 0.05).

### Morphological and hemodynamic parameters of the draining veins

In terms of morphological parameters, no significant differences were found between the ruptured and unruptured AVMs, including inlet area, exit area, absolute length, actual length, and tortuosity index. However, unruptured AVMs have a higher outlet/inlet cross-sectional area ratio [0.74 (0.63, 1.26) vs. 0.29 (0.25, 0.35), *p* = 0.045] (Table 2).

**Table 2:** Morphological and quantitative hemodynamic parameters of the AVM draining veins.

Characteristics	Ruptured	Unruptured	*p*-value
No. of patients	6	5	
Inlet area, mm^2^ (IQR)	22.87 [10.93, 68.90]	29.86 [22.36, 39.17]	0.855
Exit area, mm^2^ (IQR)	9.94 [4.59, 19.40]	14.18 [14.04, 32.92]	0.201
Outlet/inlet cross-sectional area ratio (IQR)	0.29 [0.25, 0.35]	0.74 [0.63, 1.26]	0.045^*^
Absolute length, mm (IQR)	30.54 [20.40, 42.48]	21.00 [13.82, 34.60]	0.465
Actual length, mm (IQR)	63.29 [36.11, 68.58]	71.20 [42.10, 80.12]	0.715
Tortuosity index (IQR)	1.50 [1.40, 2.12]	2.06 [1.15, 3.22]	>0.999
WSS average, Pa (IQR)	0.43 [0.38, 0.59]	0.13 [0.12, 0.31]	0.144
WSS maximum, Pa (IQR)	6.82 [4.96, 8.16]	3.31 [1.37, 6.76]	0.100
Pressure average, Pa (IQR)	1030.34 [1022.46, 1038.46]	1005.58 [1003.40, 1010.05]	0.006^*^
Pressure maximum, Pa (IQR)	1054.35 [1045.95, 1074.67]	1022.40 [1019.33, 1035.94]	0.045^*^
Number of segments with high velocity (%)	36 (40.0), *n* = 90	11 (14.7), *n* = 75	<0.001^*^

AVM, arteriovenous malformation. Values are expressed as number of cases (%) or median ± IQR, unless otherwise indicated. ^*^Statistical significance (*p* < 0.05).

In comparing quantitative hemodynamic parameters, the WSS average and maximum in each patient were similar between the ruptured and unruptured AVMs (*p* = 0.144, *p* = 0.100, respectively) (Table 2). However, the ruptured AVMs showed significantly higher WSS in the posterior segment [C segment, 0.74 (0.69, 1.25) vs. 0.16 (0.10, 0.37) Pa, *p* = 0.045] (Table 3). In terms of intravascular pressure, the ruptured AVMs appeared significantly higher pressure average [1030.34 (1022.46, 1038.46) vs. 1005.58 (1003.40, 1010.05) Pa, *p* = 0.006], and higher pressure maximum [1054.35 (1045.95, 1074.67) vs. 1022.4 (1019.33, 1035.94) Pa, *p* = 0.045]. In the subsegment analysis, the pressure of each segment of the drainage vein of ruptured AVMs was higher than that of unruptured AVMs in the same segment (A segment: *p* = 0.011, B segment: *p* = 0.006, C segment: *p* = 0.006, respectively). In terms of velocity, ruptured AVMs showed more high-velocity segments than unruptured AVMs [ruptured vs. unruptured: 36 (40%) vs. 11 (14.7%), *p* = 0.037]. And the ruptured AVM tends to have high flow rates in the posterior segment (C segment: *p* = 0.011).

**Table 3:** Comparison of hemodynamic parameters between different segments of the AVM draining vein.

Characteristics	Ruptured	Unruptured	*p*-value
No. of patients	6	5	
Pressure in each segment, Pa (IQR)			
AP [median (IQR)]	1039.28 [1031.07, 1048.18]	1008.17 [1006.68, 1017.49]	0.011^*^
BP [median (IQR)]	1030.61 [1023.59, 1039.78]	1005.55 [1004.43, 1009.50]	0.006^*^
CP [median (IQR)]	1015.30 [1009.44, 1021.97]	1001.67 [1001.47, 1002.77]	0.006^*^
WSS in each segment, Pa (IQR)			
AWSS [median (IQR)]	0.26 [0.20, 0.31]	0.16 [0.13, 0.29]	0.584
BWSS [median (IQR)]	0.47 [0.32, 0.53]	0.14 [0.10, 0.37]	0.201
CWSS [median (IQR)]	0.74 [0.69, 1.25]	0.16 [0.10, 0.37]	0.045^*^
Velocity in each segment, m/s (IQR)			
AV [median (IQR)]	0.04 [0.03, 0.04]	0.02 [0.01, 0.03]	0.273
BV [median (IQR)]	0.05 [0.03, 0.06]	0.02 [0.01, 0.04]	0.201
CV [median (IQR)]	0.08 [0.07, 0.09]	0.02 [0.01, 0.04]	0.011^*^

AVM, arteriovenous malformation. Values are expressed as number of cases (%) or Median ± IQR, unless otherwise indicated. ^*^Statistical significance (*p* < 0.05).

### Spatial distribution of WSS and intravascular pressure in the draining veins with segment

**Figure 3:**
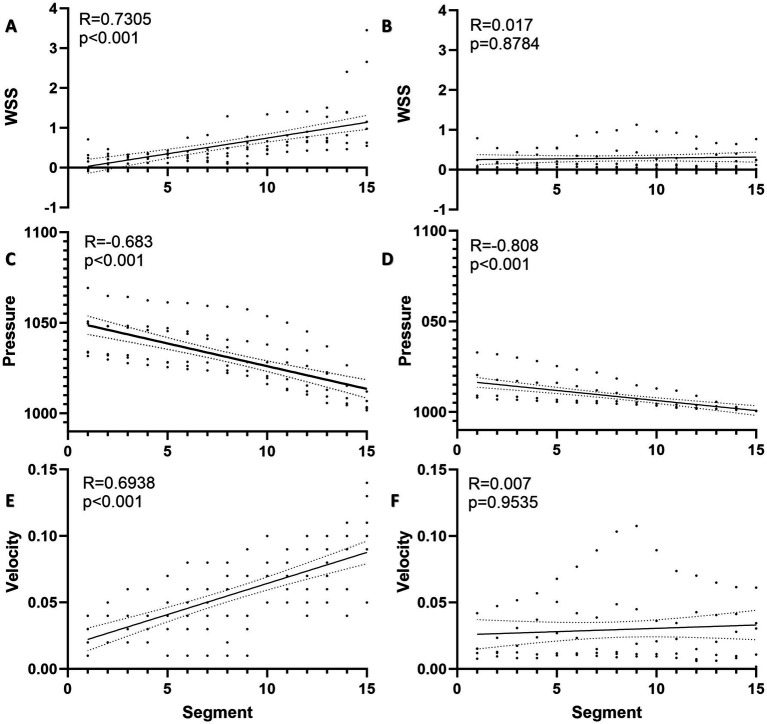
Spatial distribution of hemodynamics in AVM draining vein. **(A)** The WSS of ruptured AVMs showed a positive linear correlation with segmenting. **(B)** The WSS of unruptured AVMs and segments was no linear correlation. **(C)** The pressure of ruptured AVMs showed a negative linear correlation with segmenting. **(D)** The pressure of unruptured AVMs showed a stronger negative linear correlation with segmenting. **(E)** The flow velocity of ruptured AVMs showed a positive linear correlation with segmenting. **(F)** The flow velocity of unruptured AVMs and segments was no linear correlation.

The WSS of ruptured AVMs showed a positive linear correlation with segmenting (*R* = 0.731, *p* < 0.001), whereas this phenomenon was not observed in the unruptured AVMs (*R* = 0.017, *p* = 0.8784) (Figures 3A,B). And the pressure of both ruptured and unruptured AVMs showed negative linear correlations with segmenting (ruptured: *R* = −0.683, *p* < 0.001; unruptured: *R* = −0.808, *p* < 0.001) (Figures 3C,D). The mean blood flow velocity of ruptured AVMs showed a positive linear correlation with segmenting (*R* = 0.6938, *p* < 0.001), whereas this phenomenon was not observed in the unruptured AVMs (*R* = 0.007, *p* = 0.9535) (Figures 3E,F).

## Discussion

In this study, we initiated a preliminary CFD-based study to explore the hemodynamic characteristics of rupture in the AVM-draining veins. We found that the intravascular pressure of draining veins in ruptured AVMs was significantly higher than those in unruptured AVMs, and the WSS of the posterior segment was higher in ruptured AVMs. Moreover, the WSS of draining veins in ruptured AVMs showed a linear increase trend with segmenting, and ruptured AVMs were more likely to be accompanied by high-velocity segments in the draining vein. These results imply that the hemodynamics within the draining vein of ruptured AVMs are often in a more intense and unstable state, and the findings of this study contribute to our understanding of the mechanisms of interaction between the hemodynamics and angioarchitecture characteristics in the AVM-draining vein.

Many previous studies have proposed that venous drainage abnormalities are strongly associated with hemorrhagic events in AVMs (16, 17). Draining venous stenosis and exclusive deep venous drainage appeared to be independent predictors of hemorrhagic presentation (5). Occlusive hyperemia due to stenosis of the draining vein and flow stasis caused by rapid inflow and slow outflow were recognized as the underlying mechanisms of AVM rupture (9, 11). Besides, the main pathophysiological mechanism of rupture caused by deep venous drainage has been linked to higher outflow impedance (18). However, current research lacks an exploration of the hemodynamic mechanisms characteristic of draining veins associated with AVM rupture. This study area presents several challenging issues, such as the difficulty in obtaining individualized boundary conditions for draining veins and the more irregular vascular morphology of veins compared to arteries.

The model-based CFD calculation has been used as a reliable modality to investigate blood flow patterns in cerebral vascular diseases, and the quantitative calculation of WSS and other parameters by CFD shows a convincing fidelity (19). However, CFD has not resulted in satisfactory progress in AVMs, since the exact geometry of the AVM compartments still eludes current imaging methods (20). Therefore, CFD hemodynamic analysis based on feeding artery or draining vein becomes a feasible way to indirectly explore the biological behavior of AVMs. In this study, based on the important role of AVM draining vein in rupture found in previous studies, we focused on the hemodynamic analysis of AVM draining vein through CFD. Finally, we found that the intravascular pressure of draining veins in ruptured AVMs was significantly higher than those in unruptured AVMs, and the WSS of the posterior segment was higher in ruptured AVMs. This partially contradicts the hypothesis proposed by Alqadi et al. (21), which suggested that decreased WSS leads to draining vein stenosis and subsequently promotes hemorrhage. We believe this discrepancy may be due to differences in the mechanisms of AVM rupture. Alqadi’s et al. (21) findings might only apply to AVMs complicated by draining vein stenosis, whereas in our study, we better balanced this angioarchitectural variable. Moreover, the impact of WSS on vascular architecture might be bidirectional (22). The effect of WSS on angioarchitecture may be bidirectional. Previous studies reported that intima hyperplasia may be associated with low WSS, whereas destruction and thinning of vascular wall structure may be associated with high WSS (7, 23). In this study, we found a significant increase in draining vein WSS and more high-velocity segments in ruptured AVMs, implying the presence of more turbulent separated flow (Reynolds number = 12,429) and more deviated laminar flowing the draining veins of ruptured AVMs.

The correlation between draining vein pressure and rupture in AVMs has not been elucidated. Zhang et al. (12) demonstrated higher feeding artery pressure and overloaded pressure across the nidus were associated with AVM rupture. Miyasaka et al. (24) found higher drainage venous pressure in ruptured AVMs by intravascular pressure measurement. The present study found a similar phenomenon to the above study, and a linear decreased draining veins’ pressure in the unruptured AVMs, which further indicates that the hemodynamics in the draining vein in unruptured AVMs were more stable. Generally, the vein walls are thought to thicken with increased pressure (25), intravascular pressure can affect endothelial cytoplasmic swelling, cell deformation, cell disintegration, and subsequent erosion, finally contributing to local thrombosis (26). Because of the hemodynamically associated endovascular changes in the endothelium, neointimal hyperplasia may cause the veins to narrow and/or occlude (22). On the other hand, the variation in vessel wall parameters increases turbulence and introduces flow separation, leading to damage repair and reconstruction of the endothelial cell (27). Therefore, the hemorrhagic presentation in AVMs may result from the interaction of hemodynamics and specific angioarchitecture resulting in occlusive congestion within the AVM nidus (9).

There were some limitations in our study. Firstly, the sample size was small, and individual heterogeneity may seriously affect the measurement results of hemodynamic parameters. Second, because of the unavailability of the pulse curve of the draining veins, we used a simplified steady-state CFD model for blood flow simulation instead of the transient model. Thus, we are not available for hemodynamic parameters such as oscillatory shear index (OSI) and relative resident time (RRT) calculated by transient models. However, we used the QDSA to calculate and extract the initial flow velocity of the individual draining vein, which allowed us to perform hemodynamic simulations in a more realistic scenario. Third, this study is a preliminary hemodynamic analysis of the main drainage vein of supratentorial AVM, without considering the influence of other secondary drainage veins. However, regardless of the mentioned problems, this study could preliminarily clarify the characteristics of hemodynamic distribution in AVM drainage veins with different clinical manifestations.

## Conclusion

The current study demonstrated that the hemodynamics of draining veins might be associated with AVM rupture. The intravascular pressure of draining veins in ruptured AVMs was significantly higher than the unruptured AVMs, and the WSS of the posterior segment was higher in ruptured AVMs. The WSS of draining veins in ruptured AVMs showed a linear increase trend with segmenting, and ruptured AVMs were more likely to be accompanied by high-velocity segments in the draining vein.

## Data Availability

The datasets presented in this article are not readily available because all original data are available upon reasonable request to the corresponding author. Requests to access the datasets should be directed to chenxiaolin@bjtth.org.

## References

[1] Lawton MT Rutledge WC Kim H Stapf C Whitehead KJ Li DY. Brain arteriovenous malformations. Nat Rev Dis Primers. (2015) 1:15008. doi: 10.1038/nrdp.2015.8 27188382

[2] Furlan AJ Whisnant JP Elveback LR. The decreasing incidence of primary intracerebral hemorrhage: a population study. Ann Neurol. (1979) 5:367–73. doi: 10.1002/ana.410050410 375807

[3] Al-Shahi R Warlow C. A systematic review of the frequency and prognosis of arteriovenous malformations of the brain in adults. Brain. (2001) 124:1900–26. doi: 10.1093/brain/124.10.1900, PMID: 11571210

[4] Kim H Al-Shahi Salman R McCulloch CE Stapf C Young WL For the MARS Coinvestigators. Untreated brain arteriovenous malformation: patient-level meta-analysis of hemorrhage predictors. Neurology. (2014) 83:590–7. doi: 10.1212/WNL.0000000000000688, PMID: 25015366 PMC4141996

[5] Feghali J Yang W Xu R Liew J McDougall CG Caplan JM. R2eD AVM score. Stroke. (2019) 50:1703–10. doi: 10.1161/STROKEAHA.119.025054, PMID: 31167618

[6] Chen Y Han H Meng X Jin H Gao D Ma L. Development and validation of a scoring system for hemorrhage risk in brain arteriovenous malformations. JAMA Netw Open. (2023) 6:e231070. doi: 10.1001/jamanetworkopen.2023.1070, PMID: 36857052 PMC9978947

[7] Shakur SF Amin-Hanjani S Mostafa H Charbel FT Alaraj A. Hemodynamic characteristics of cerebral arteriovenous malformation feeder vessels with and without aneurysms. Stroke. (2015) 46:1997–9. doi: 10.1161/STROKEAHA.115.009545, PMID: 25991417

[8] Chang W Loecher MW Wu Y Niemann DB Ciske B Aagaard-Kienitz B. Hemodynamic changes in patients with arteriovenous malformations assessed using high-resolution 3D radial phase-contrast MR angiography. AJNR Am J Neuroradiol. (2012) 33:1565–72. doi: 10.3174/ajnr.A3010, PMID: 22499844 PMC6278605

[9] Lin TM Yang HC Lee CC Wu HM Hu YS Luo CB. Stasis index from hemodynamic analysis using quantitative DSA correlates with hemorrhage of supratentorial arteriovenous malformation: a cross-sectional study. J Neurosurg. (2019) 132:1574–82. doi: 10.3171/2019.1.JNS183386 31026828

[10] Al-Rodhan NRF Sundt TM Piepgras DG Nichols DA Rßfenacht D Stevens LN. Occlusive hyperemia: a theory for the hemodynamic complications following resection of intracerebral arteriovenous malformations. J Neurosurg. (1993) 78:167–75. doi: 10.3171/jns.1993.78.2.0167, PMID: 8421198

[11] Alexander MD Cooke DL Nelson J Guo DE Dowd CF Higashida RT. Association between venous angioarchitectural features of sporadic brain arteriovenous malformations and intracranial hemorrhage. AJNR Am J Neuroradiol. (2015) 36:949–52. doi: 10.3174/ajnr.A4224, PMID: 25634722 PMC4433780

[12] Zhang Y Chen Y Li R Ma L Han H Li Z. Overloaded transnidal pressure gradient as the hemodynamic mechanism leading to arteriovenous malformation rupture: a quantitative analysis using intravascular pressure monitoring and color-coded digital subtraction angiography. J Neurointerv Surg. (2024):jnis-2023-021348. doi: 10.1136/jnis-2023-021348, PMID: 38471763

[13] DeGroff CG Shandas R Valdes-Cruz L. Analysis of the effect of flow rate on the Doppler continuity equation for stenotic orifice area calculations: a numerical study. Circulation. (1998) 97:1597–605. doi: 10.1161/01.CIR.97.16.1597, PMID: 9593565

[14] Larrabide I Kim M Augsburger L Villa-Uriol MC Rüfenacht D Frangi AF. Fast virtual deployment of self-expandable stents: method and in vitro evaluation for intracranial aneurysmal stenting. Med Image Anal. (2012) 16:721–30. doi: 10.1016/j.media.2010.04.009, PMID: 20627664

[15] Atkinson RP Awad IA Batjer HH Dowd CF Furlan A Giannotta SL. Reporting terminology for brain arteriovenous malformation clinical and radiographic features for use in clinical trials. Stroke. (2001) 32:1430–42. doi: 10.1161/01.STR.32.6.1430, PMID: 11387510

[16] Nataf F Meder JF Roux FX Blustajn J Merienne L Merland JJ. Angioarchitecture associated with haemorrhage in cerebral arteriovenous malformations: a prognostic statistical model. Neuroradiology. (1997) 39:52–8. doi: 10.1007/s002340050367, PMID: 9121650

[17] Duong DH Young WL Vang MC Sciacca RR Mast H Koennecke HC. Feeding artery pressure and venous drainage pattern are primary determinants of hemorrhage from cerebral arteriovenous malformations. Stroke. (1998) 29:1167–76. doi: 10.1161/01.STR.29.6.1167 9626290

[18] Kellner CP McDowell MM Phan MQ Connolly ES Lavine SD Meyers PM. Number and location of draining veins in pediatric arteriovenous malformations: association with hemorrhage. J Neurosurg Pediatr. (2014) 14:538–45. doi: 10.3171/2014.7.PEDS13563, PMID: 25238624 PMC9879622

[19] Gharahi H Zambrano BA Zhu DC DeMarco JK Baek S. Computational fluid dynamic simulation of human carotid artery bifurcation based on anatomy and volumetric blood flow rate measured with magnetic resonance imaging. Int J Adv Eng Sci Appl Math. (2016) 8:40–60. doi: 10.1007/s12572-016-0161-6, PMID: 27546999 PMC4987097

[20] Steiger HJ. Recent progress understanding pathophysiology and genesis of brain AVM-a narrative review. Neurosurg Rev. (2021) 44:3165–75. doi: 10.1007/s10143-021-01526-0, PMID: 33837504 PMC8592945

[21] Alqadi M Brunozzi D Linninger A Amin-Hanjani S Charbel FT Alaraj A. Cerebral arteriovenous malformation venous stenosis is associated with hemodynamic changes at the draining vein-venous sinus junction. Med Hypotheses. (2019) 123:86–8. doi: 10.1016/j.mehy.2019.01.003, PMID: 30696602 PMC6370035

[22] Shakur SF Hussein AE Amin-Hanjani S Valyi-Nagy T Charbel FT Alaraj A. Cerebral arteriovenous malformation flow is associated with venous intimal hyperplasia. Stroke. (2017) 48:1088–91. doi: 10.1161/STROKEAHA.116.015666 28235957

[23] Zarins CK Giddens DP Bharadvaj BK Sottiurai VS Mabon RF Glagov S. Carotid bifurcation atherosclerosis. Quantitative correlation of plaque localization with flow velocity profiles and wall shear stress. Circ Res. (1983) 53:502–14. doi: 10.1161/01.RES.53.4.502 6627609

[24] Miyasaka Y Kurata A Tokiwa K Tanaka R Yada K Ohwada T. Draining vein pressure increases and hemorrhage in patients with arteriovenous malformation. Stroke. (1994) 25:504–7. doi: 10.1161/01.STR.25.2.504 8303764

[25] Lee T Wadehra D. Genetic causation of neointimal hyperplasia in hemodialysis vascular access dysfunction. Semin Dial. (2012) 25:65–73. doi: 10.1111/j.1525-139X.2011.00967.x, PMID: 21917012 PMC3965266

[26] Fry DL. Acute vascular endothelial changes associated with increased blood velocity gradients. Circ Res. (1968) 22:165–97. doi: 10.1161/01.RES.22.2.165, PMID: 5639037

[27] Javid Mahmoudzadeh Akherat SM Cassel K Boghosian M Hammes M Coe F. A predictive framework to elucidate venous stenosis: CFD & shape optimization. Comput Methods Appl Mech Eng. (2017) 321:46–69. doi: 10.1016/j.cma.2017.03.036, PMID: 28649146 PMC5479643

